# Protein engineering of NADH pyrophosphatase for efficient biocatalytic production of reduced nicotinamide mononucleotide

**DOI:** 10.3389/fbioe.2023.1159965

**Published:** 2023-04-04

**Authors:** Ye Liu, Jin-Song Gong, George Marshall, Chang Su, Michael Hall, Heng Li, Guo-Qiang Xu, Jin-Song Shi, Zheng-Hong Xu

**Affiliations:** ^1^ Key Laboratory of Carbohydrate Chemistry and Biotechnology, Ministry of Education, School of Life Sciences and Health Engineering, Jiangnan University, Wuxi, China; ^2^ Yixing Institute of Food and Biotechnology Co., Ltd., Yixing, China; ^3^ Seragon Biosciences, Inc., Irvine, CA, United States; ^4^ National Engineering Research Center for Cereal Fermentation and Food Biomanufacturing, School of Biotechnology, Jiangnan University, Wuxi, China

**Keywords:** reduced nicotinamide mononucleotide, NADH pyrophosphatase, expression, semi-rational engineering, protein engineering

## Abstract

**Introduction:** NADH pyrophosphatase, a hydrolase catalyzing the phosphate bond of NADH to reduced nicotinamide mononucleotide, has potential applications in the food, cosmetic and pharmaceutical industry.

**Methods:** Here, we investigated the effects of vector screening, promoter and RBS strategies on NADH pyrophosphatase expression and protein engineering on its enzymatic activity and thermal stability.

**Results:** In this study, we describe a NADH pyrophosphatase derived from *Escherichia coli* (*EcNudc*). Strategies focusing on expression regulation including screening vectors, optimizing promoters and ribosome binding sites were utilized to enhance the productivity of *EcNudc* (1.8 U/mL). Moreover, protein engineering was adopted to further improve the catalytic properties of *EcNudc*, achieving 3.3-fold higher activity and 3.6-fold greater thermostability at 50°C. Furthermore, fermentation for the combined mutant R148A-H149E (*EcNudc-M*) production in a 7 L fermenter was implemented and the enzyme activity of *EcNudc-M* reached 33.0 U/mL. Finally, the *EcNudc-M* was applied in the catalysis of NADH with the highest NMNH yield of 16.65 g/L.

**Discussion:** In conclusion, we constructed a commercially available genetically engineered strain with high activity and thermal stability of NADH pyrophosphatase, laying a broad foundation for the biocatalytic industrial production of NMNH and expand its application range.

## 1 Introduction

NAD^+^, also known as coenzyme I, is involved in multiple metabolic pathways in organisms, including the tricarboxylic acid cycle, glycolysis, and fatty acid oxidation (Hikosaka et al., 2019) along with regulation of cellular functions such as DNA repair and cellular senescence (Covarrubias et al., 2021). Studies have shown that NAD^+^ can activate mitochondrial function to mitigate neurodegenerative diseases such as Alzheimer’s disease (AD) and Parkinson’s disease (PD) (Hikosaka et al., 2019), to enhance sirtuin activity in the treatment of heart and kidney diseases (Hershberger et al., 2017) and to stimulate Fndc5/irisin factor to relieve non-alcoholic fatty liver disease (Li et al., 2021).

NAD^+^ levels in organisms decrease with age. Supplementation with NAD^+^ precursors, such as nicotinic acid (NA), nicotinamide, nicotinamide riboside (NR), and nicotinamide mononucleotide (NMN), can be used as an effective way to increase NAD^+^ levels. However, NA activates the Gpr109A receptor leading to flushing and nicotinamide may inhibit sirtuin function and affect reverse cholesterol transport (Bogan and Brenner, 2008). Therefore, NR and NMN are considered to the leading products for efficacy. Due to the high-doses dependent limitation of NMN and NR, the reduced nicotinamide mononucleotide (NMNH) has recently attracted increasing attention as a stronger NAD^+^ enhancer (Liu et al., 2021; Zapata-Perez et al., 2021).

Through metabolomic analysis and *in vitro* experiments, Liu et al. demonstrated that NMNH inhibited glycolysis, the tricarboxylic acid cycle and cell growth (Liu et al., 2021). Zapata-Pérez et al. supplemented the same concentrations of NMN and NMNH in different mouse and human cell lines, respectively, and found that NMNH significantly enhanced the content of NAD^+^ and was more effective than NMN. In addition, by injecting 250 mg/kg NMN and NMNH into C57BL/6N mice, it was found that NMNH maintained NAD^+^ levels for a longer period of time than NMN. Furthermore, NMNH supplementation also protected renal tubular epithelial cells from hypoxia and reoxygenation injury (Zapata-Perez et al., 2021).

**FIGURE 1:**
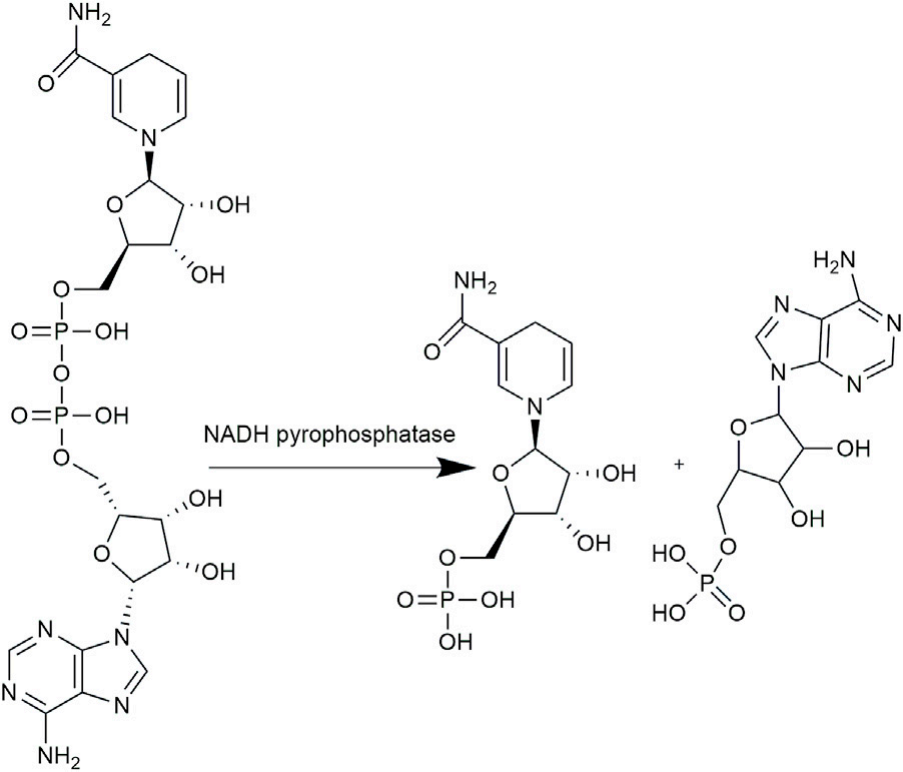
Biosynthesis of NMNH from NADH.

At present, there are two main methods for the synthesis of NMNH. The chemical reduction method reduces NMN into NMNH using reducing agent thiourea dioxide (TDO). Liu et al. (2021) reacted 340 mg NMN and 125 mg TDO in 10% ammonia solution at 40°C for 1 h. The product NMNH was then purified using preparative liquid phase and concentrated by vacuum drying. However, the reducing agent TDO is irritating to skin and mucous membranes, and may easily cause explosive decomposition under heat and impact conditions. The biological catalytic method depends on the catalysis of NADH pyrophosphatase (*Nudc*), which converts NADH into equal mole equivalent of NMNH and AMP (Figure 1). Compared with chemical method, the enzymatic synthesis reaction system is performed under mild reaction conditions with eco-friendly nature and shows high substrate specificity. NADH pyrophosphatase belongs to the Nudix hydrolase superfamily with the conserved sequence GX_5_EX_7_REVXEEXGU. In 1994, Frick and Bessman (1995) expressed *Escherichia coli* NADH pyrophosphatase in *E. coli* MG1655 strain. In catalyzing the hydrolysis of a series of dinucleotide pyrophosphates, the enzyme has a particular preference for NADH and the *V*_max_ reached 7.6 µ/mg. In addition, other NADH pyrophosphatases have also been discovered and characterized in different organisms. For example, AbdelRaheim et al. (2001) found that the NADH diphosphatase encoded by *Saccharomyces cerevisiae* NPY1 Nudix hydrolase gene in peroxisome was successfully expressed and purified in *E. coli* BL21 (DE3) and the *V*_max_ reached 2.0 μmol/min/mg. Wang et al. (2011) expressed the *Nudc* genes from *M. tuberculosis* H37Rv (*NudC*_*Rv*_) and *M. bovis* BCG (*NudC*_*BCG*_) in *E. coli* BL21 (DE3). In the presence of Mg^2+^, the specific enzyme activities were 1.59 μ/mg and 0.02 μ/mg, respectively. Moreover, human NUDT12 and mouse NUDT13 were recombinantly expressed in insect cells by Abdelraheim et al. (2003; 2017).

Although various *Nud*cs have been cloned and expressed, their properties and productivities still cannot meet the industrial applications, and there are few studies focusing on enzyme modification. In this study, we first expressed the *Nudc* in *E. coli* BL21 (DE3) (named *EcNudc*). In order to enhance the expression of *EcNudc*, a series of regulation strategies including optimization of the vector, promoter engineering and ribosome binding site (RBS) engineering were conducted. Furthermore, site-directed mutation was performed to enhance the catalytic activity and thermal stability of *EcNudc*. To assess the catalytic potential of NADH pyrophosphatase, fed-batch fermentation in a 7 L fermenter was performed. Finally, the NADH biocatalytic synthesis of NMNH reaction process was also evaluated to investigate industrial application potential. Overall, we constructed a commercially available genetically engineered strain with high activity and thermal stability of NADH pyrophosphatase, laying a broad foundation for biosynthetic NMNH application aspects.

## 2 Materials and methods

### 2.1 Materials

*E. coli* JM109 was used for gene cloning and *E. coli* BL21 (DE3) was used for recombinant protein expression, respectively. The expression plasmids including pET-28a (+), pET-3b, pRSFDuet-1, pCDFDuet-1, pACYCDuet-1 are all kept in the laboratory. The sequence of *EcNudc* was optimized and modified based on NADH pyrophosphatase from *E. coli* (GenBank: NEY29540.1).

The shake flask fermentation medium included 10 g/L tryptone, 5 g/L yeast extract, 10 g/L NaCl. The seed medium included (g/L) (Gong et al., 2018): tryptone 20, yeast extract 5, KCl 0.19, NaCl 0.5, glucose 3.6, MgCl_2_ 0.95, trace elements (FeCl_3_.6H_2_O 6, CuCl_2_.2H_2_O 0.2, CaCl_2_ 0.2, EDTA 0.5, ZnSO_4_.7H_2_O 0.58, MnSO_4_·H_2_O 0.3) 2 mL/L. The on-tank fermentation medium included (g/L) (Gong et al., 2018): tryptone 20, yeast extract 5, KCl 0.19, NaCl 0.5, glucose 10, MgCl_2_ 0.95, trace elements 2 mL/L. The feeding medium contained (g/L): glucose 700, MgSO_4_ 14, trace elements 14 mL/L.

### 2.2 Engineering of promoters and RBSs for improving *EcNudc* expression

The promoters were kept in the laboratory. According to the translation rate of wild-type RBS on the plasmid calculated by the RBS calculator (Salis et al., 2009), RBS of different intensities (RBS5000-RBS50000) were designed to regulate the expression of the NADH pyrophosphatase. The RBS was replaced by inverse PCR technology. The PCR system consists of 50 μL total, including 20 μL ddH_2_O, 2 μL upstream primer, 2 μL downstream primer, 1 μL template, 25 μL 2 × Phanta Max buffer. The PCR amplification procedure was conducted as follows: 95°C for 3 min, then followed by 34 cycles (95°C for 15 s, 55°C for 15 s, 72°C for 6 min), and last extension 72°C for 5 min. The PCR products were digested with 1 μL DPNI enzyme for 2 h at 37°C (10 μL system includes 1 μL DPNI enzyme, 1 μL 10× Q.cut Buffer, 8 μL PCR product), and then transformed into *E. coli* hosts for cloning and expression. Correctly sequenced strains were subjected to fermentation. The primers are shown in Supplementary Table S1.

### 2.3 NADH pyrophosphatase assay

NADH pyrophosphatase activity was measured by determining the content of inorganic phosphate. The standard reaction mixture (50 μL) consisted of 5 mM MgCl_2_, 50 mM Tris-HCl, pH = 8.0; 25 mM NADH; 4 U alkaline phosphatase (calf intestine) and enzyme. After reaction with 37°C for 15 min, 250 μL of 4 mM EDTA was added to terminate the reaction. And then inorganic phosphate was measured by adding 700 μL of mixture (10% ascorbic acid and 0.42% ammonium molybdate tetrahydrate) at a wavelength of 820 nm (Ames and Dubin, 1960). One unit of NADH pyrophosphatase hydrolyzes 1 μmol NADH per minute under these conditions.

### 2.4 Site-directed mutagenesis of *EcNudc*

Through HotSpot Wizard web service, we selected eight residues (Sumbalova et al., 2018). The NADH pyrophosphatase site was mutated using inverse PCR. The method is the same as above.

### 2.5 Protein expression, purification of *EcNudc* and mutant, and kinetic parameter analysis

The recombinant strains and mutants were first cultivated at 37°C for 2 h. When OD_600_ reached 0.6–0.8, adding 0.5 mM IPTG to induce the expression of the strain at 25°C for 12 h. After fermentation, recombinant cells were harvested by centrifugation at 8,000 rpm, 4°C for 10 min. Each cell was washed and resuspended with Tris-HCl (PH = 8.0). After the resuspension was sonicated for 30 min, the supernatant was obtained by centrifugation at 8,000 rpm for 10 min at 4°C. Ni^2+^ column affinity chromatography was used for protein purification and sodium dodecyl sulfate-polyacrylamide gel electrophoresis (SDS-PAGE) was used to assay protein purification. Protein concentration was determined by BCA Protein Assay Kit. Kinetic parameters were determined for *EcNudc* and mutant strains by using different substrate concentrations (0.25–10 mM).

### 2.6 Molecular docking analysis

The protein structure model was built by SWISS-MODEL (Bienert et al., 2017; Waterhouse et al., 2018). Molecular docking was performed using AutoDock Vina (Trott and Olson, 2010; Eberhardt et al., 2021). The interactions between ligands and receptors were analyzed accordingly.

### 2.7 Fermentation experiments on tanks

The single colony of *EcNudc-M* from an agar plate (kanamycin) was selected into 10 mL of LB medium and cultivated 37°C and 220 rpm for 12 h. Then transferred to seed medium at 2% inoculum and cultivated at the same condition. Finally, the seed solution was then inoculated at a 10% inoculum into a 7 L fermenter with 4 L of medium, and when OD_600_ = 2, 0.1 mM IPTG was added and induced at 25°C. When the glucose concentration was below 2 g/L, the constant rate feeding was started at a rate of 0.1 mL/min. The speed and ventilation were regulated throughout the process to keep the dissolved oxygen above 10%, and the pH = 7.0 by adding ammonia.

### 2.8 Catalytic performance of the mutant strain and HPLC analysis

The catalytic performance of NADH pyrophosphatase for the bioconversion of NADH to NMNH was investigated. The supernatant of recombinant *E. coli* NADH pyrophosphatase was prepared and subjected to biotransformation reactions. The reaction solution was periodically sampled to detect the concentration of substrate NADH and product NMNH by HPLC. Using Diamonsil C18 column (5 μm, 250 × 4.6 mm) to perform HPLC detection at 340 nm. The mobile phase consists of two parts. Mobile phase A was methanol, water, acetic acid and tetrabutylammonium hydroxide (3/97/1/0.6), mobile phase B was methanol. The procedure was as follows: 0 min, 100% A; 5 min, 100% A; 25 min, 40% A; 30 min, 20% A; 40 min, 100% A.

## 3 Results and discussion

### 3.1 Screening of plasmids overexpressing *Ec Nudc*

**FIGURE 2:**
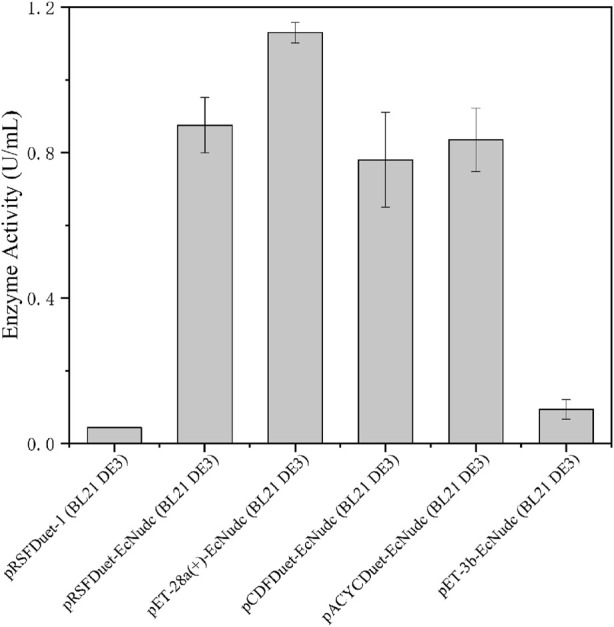
Screening of *EcNudc* expression vectors.

*E. coli* is widely used as a host for protein expression due to its simple genetic manipulation and rapid growth. Plasmids with different copy numbers are important factors affecting protein expression (Hayat et al., 2018). Therefore, we performed the initial expression of the NADH pyrophosphatase gene in *E. coli* and the enzyme activity of strain pRSFDuet-EcNudc/BL21 (DE3) was 0.88 U/mL, which was a high copy number plasmid (Shoji et al., 2021). SDS-PAGE analysis showed that the enzyme was successfully expressed in *E. coli* without inclusion body formation (Supplementary Figure S1). Then, plasmids with other copy numbers and expression patterns were also used to probe the effect on enzyme activity (Hayat et al., 2018). As shown in Figure 2, the pET-28a (+)-EcNudc/BL21 (DE3) strain had the highest enzyme activity, reaching 1.1 U/mL. In contrast, for the strain pRSFDuet-EcNudc carrying a high copy number plasmid, the enzyme activity was about the same as that of the strain pACYCDuet-EcNudc carrying a low copy number plasmid. This also indicates that the enzyme activity is not linearly correlated with the copy number (Shoji et al., 2021). Therefore, the pET-28a (+)-EcNudc/BL21 (DE3) strain was selected for further research.

### 3.2 Engineering of promoter and RBS sequences to enhance the enzyme activity of *EcNudc*

**FIGURE 3:**
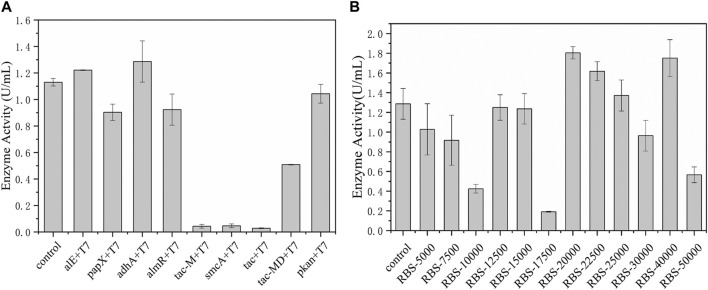
Adaptability modification of promoters and RBSs. **(A)** Adaptability modification of promoters. **(B)** Adaptability modification of RBSs with strengths.

The promoters and ribosome binding sites (RBSs) are important elements for regulating gene transcription and the initiation rate of protein translation, respectively. Optimizing promoters and RBS sequences have become an effective strategy to rationally improve gene expression and increase productivity. Hou et al. (2013) increased the activity of D-amino acid oxidase (DAAO) by replacing the promoter and RBS, promoting its soluble expression. Duan et al. (2021) systematically studied the combined effect of promoter and RBS in *C. glutamicum* for the first time, and finally the production of arginine and citrulline in the 7 L reactor increased by 1.61 times and 2.35 times, respectively. Therefore, the promoter element was modified in the early stage. Among these promoters, adhA was the most effective in tandem with the T7 promoter and the enzyme activity reached 1.3 U/mL, increased by 18.2% (Figure 3A). On this basis, according to the RBS calculator, RBSs with different strengths were designed to further improve the catalytic ability of *EcNudc* and the constructed plasmids were transformed into *E. coli* BL21 (DE3) to compare their enzyme production levels. As shown in Figure 3B, there was no linear correlation between RBS intensity and expression, where the intensity region of 20,000–40,000 was suitable for *EcNudc* expression. The pyrophosphatase activity of RBS-20000 reached 1.8 U/mL, which was about 38.5% higher than that of the control strain. In addition, according to SDS-PAGE (Supplementary Figure S2), the expression level of RBS-20000 was increased compared with the control. Therefore, we successfully established an efficient expression system for producing NMNH in *E. coli* system.

### 3.3 Design and expression of recombinant *EcNudc* mutants through semi-rational strategies

**FIGURE 4:**
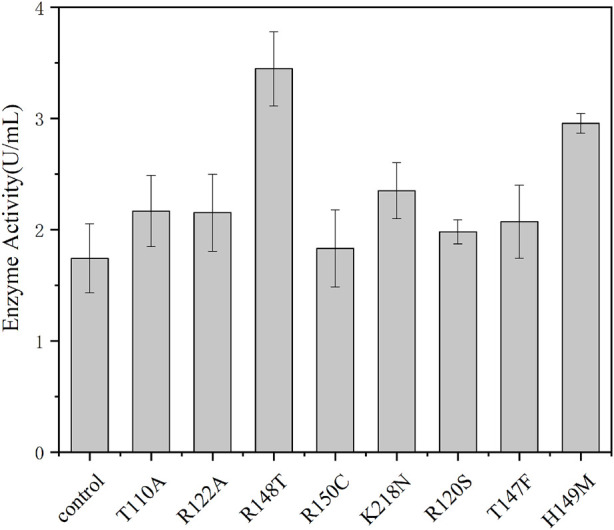
Enzyme activity of the *EcNudc* and its mutants under the standard reaction condition (37°C, pH 8.0).

It is important for industrial application to improve the catalytic capacity, thermal stability, and pH tolerance of enzymes. Thermally stable enzymes could greatly improve productivity and promote industrial applications (Lee et al., 2014; Zhu et al., 2021). HotSpot Wizard is a web server that allows semi-rational design of proteins based on bioinformatics analysis to enhance their catalytic activity, stability, etc., (Sumbalova et al., 2018). Therefore, in order to further improve the catalytic activity and thermostability of pyrophosphatase, based on the structural analysis and crucial residues prediction by HotSpot Wizard, we selected and mutated residue hotspots to the corresponding amino acids, resulting in eight mutation sites T100A, R122A, R148T, R150C, K218N, R120S, T147F, and H149M. As shown in Figure 4, the enzyme activities of R148T and H149M were significantly higher than the activity of the wild type by 91% and 64%, respectively. Therefore, two sites (R148, H149) with significantly elevated enzyme activity were subsequently subjected for site-directed saturation mutation.

### 3.4 Site-directed saturation mutagenesis of *EcNudc*

**FIGURE 5:**
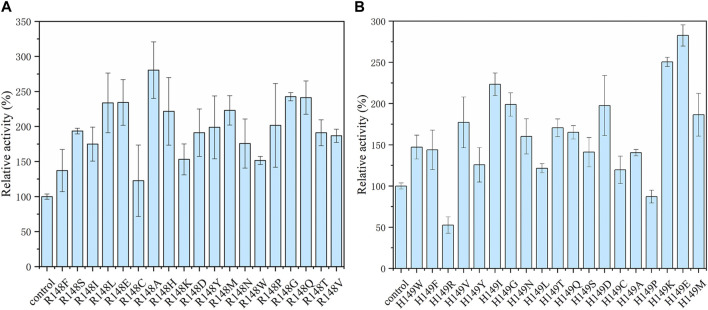
Site-directed saturation mutagenesis of *EcNudc*. **(A)** Relative activity with saturation mutations at the R148. **(B)** Relative activity with saturation mutations at the H149.

Saturation mutations were performed on the two selected residue sites. According to the results in Figure 5, the arginine at position 148 was mutated to the other 19 kinds of amino acids, and the mutant R148A achieved the most effective promotion with a 2.8-fold activity. For 149 site, the mutant H149E showed the highest activity of 5.1 U/mL. To further enhance the catalytic activity of NADH pyrophosphatase, a combined mutation of these two loci was constructed to obtain a double mutant strain R148A-H149E (named as *EcNudc-M*) with an enzyme activity of 6.0 U/mL. Multiple sequence alignment results indicate that the two sites are not fully conserved. This is the first report on these two mutant sites (Supplementary Figure S3).

**FIGURE 6:**
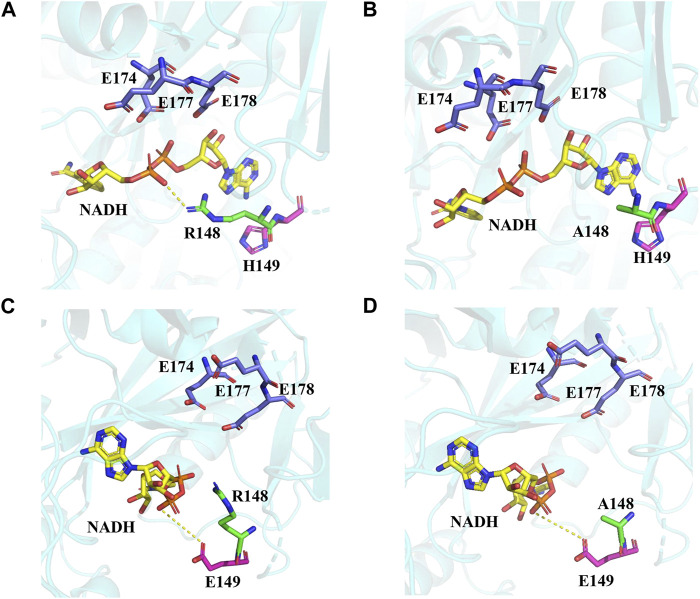
Structural analysis of the activity-related residues. **(A)** WT. **(B)** Mutant (R148A). **(C)** Mutant (H149E). **(D)** Mutant (*EcNudc-M*).

The protein structure model of *EcNudc* was built by SWISS-MODEL (Supplementary Figure S4) (Bienert et al., 2017; Waterhouse et al., 2018). The results of molecular docking showed that the R148 and H149 sites and catalytic residues (Garcia-Saura et al., 2019) (E174, E177, and E178) were on both sides of the substrate NADH. The positive charge of R148 formed an electrostatic interaction with the negative charge of the phosphate bond on the substrate NADH, hindering the contact of the phosphate bond with the catalytic residue and affecting the catalytic efficiency. Therefore, when R148 was mutated to other amino acids, the enzyme activities were all enhanced. Especially, R148A has the best performance of all variants (Figures 6A, B). The mutations in adjacent residues generally display synergistic effects (Ellis et al., 2022). When H149 was mutated to glutamic acid, it formed electrostatic repulsion with the O^−^ of the phosphate bond on NADH (Figure 6C). Therefore, the enzyme activity was enhanced. Furthermore, the enzyme activity of the combined mutant strain *EcNudc-M* reached 3.3-fold of the wild type. This is due to the loss of electrostatic interaction as well as electrostatic repulsion, which promoted the contact of the substrate NADH with the catalytic residue (Figure 6D).

### 3.5 Purification and kinetic parameter of WT and mutants

In this study, a single protein band of approximately 30 KDa appeared on SDS-PAGE after purification of WT and mutants by a nickel column (Supplementary Figure S5). Then, the kinetic parameters were measured separately. As shown in Table 1, the *K*_*m*_ value was increased from 3.5 (WT) to 7.9 (*EcNudc-M*), indicating a decreased affinity of the mutant toward NADH. However, compared with the wild type, all mutants exhibited higher catalytic efficiency. In particular, the catalytic efficiency of the mutant *EcNudc-M* was increased by 1.5-fold.

**TABLE 1:** The kinetic parameter of WT and mutants toward NADH.

Mutant	k_cat_ (s^−1^)	*K*_m_ (mM)	k_cat_/*K*_m_ (s^−1^ mM^−1^)
WT	59.5	3.5	17.2
R148A	150.3	6.8	22.1
H149E	159.0	6.1	26.0
*EcNudc-M*	208.7	7.9	26.6

### 3.6 Thermal stability assessment of mutants

**FIGURE 7:**
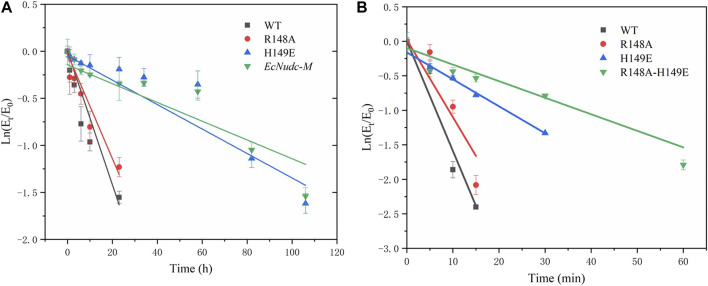
Thermal stability of wild type and mutants. **(A)** Thermal stability of wild type and mutants at 40°C. **(B)** Thermal stability of wild type and mutants at 50°C.

**FIGURE 8:**
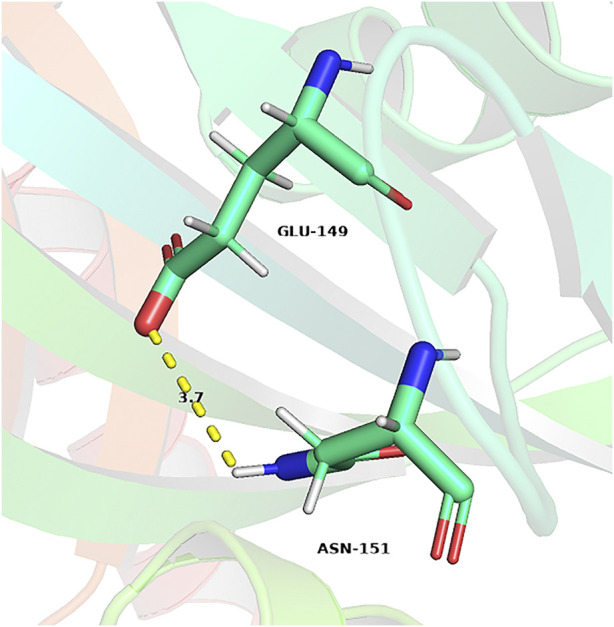
Hydrogen bonds formation between E149 and N151.

In addition to the enhanced enzyme activity, the stability of the mutant strains was also improved. As shown in Figure 7, the half-life of WT, R148A, H149E and *EcNudc-M* were 8 h, 10.9 h, 54 h, and 53.2 h at 40°C, respectively; and 4.8 min, 6.8 min, 14.1 min, and 17.5 min at 50°C, respectively. This indicated that the thermostability of *EcNudc-M* were significantly improved at 40°C and 50°C by 6.65 and 3.6 folds. The mutation of *EcNudc-M* was refinement by Rosetta relax and the enhanced stability is mainly due to the formation of hydrogen bonding between E149 and N151 (Figure 8).

### 3.7 Scale-up production of NADH pyrophosphatase in 7 L fermenter

**FIGURE 9:**
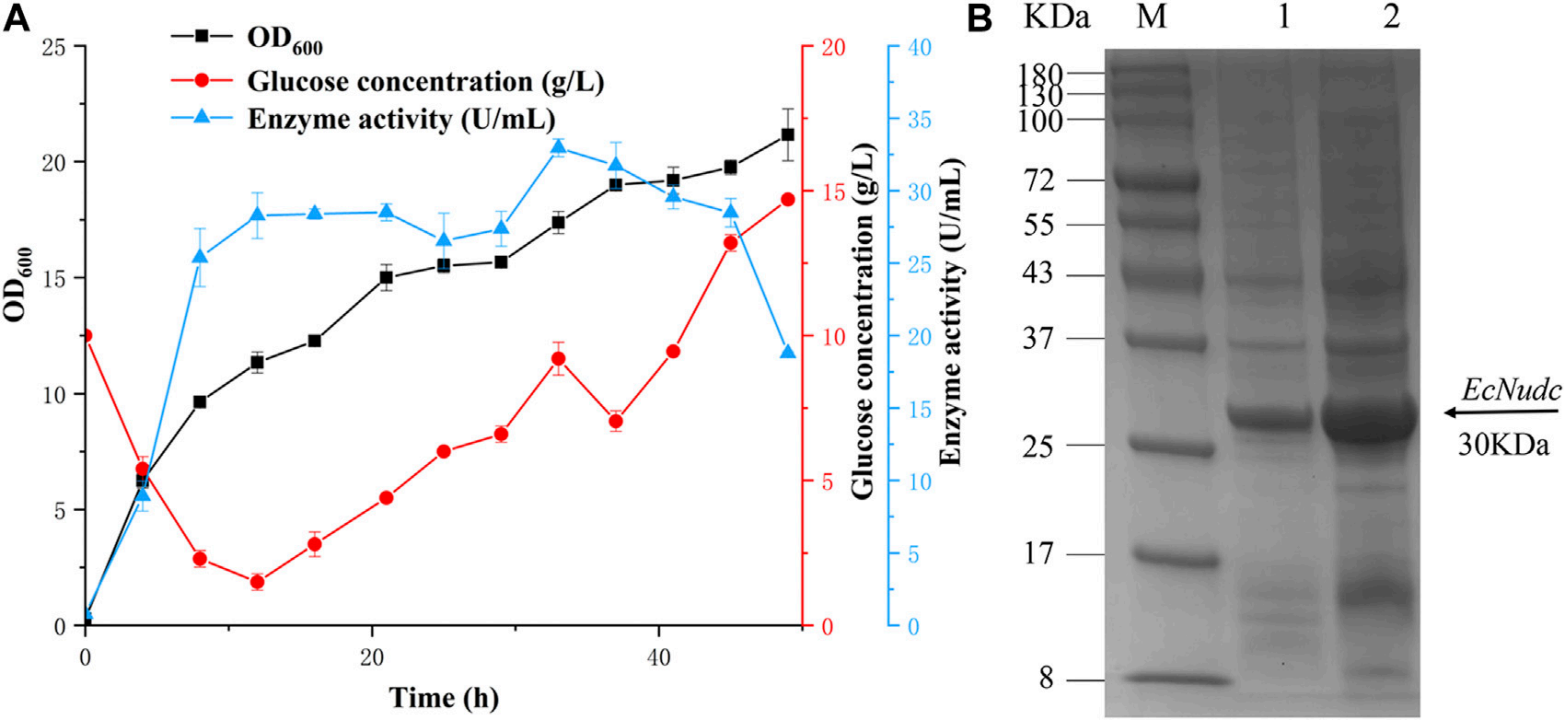
Fermentation curve of *EcNudc-M* and SDS-PAGE analysis of recombinant *EcNudc* expression. **(A)** Fermentation curve of *EcNudc-M* under constant rate replenishment strategy. **(B)** SDS-PAGE analysis of recombinant *EcNudc* expression in shake flask and fermenter level. M: protein standard marker; Lane 1: The supernatant of *EcNudc-M* in shake flask level; Lane 2: The supernatant of *EcNudc-M* in the fermenter level.

To further evaluate the production capacity of NADH pyrophosphatase, the fementation of the mutant *EcNudc-M* was expanded on a 7 L fermenter based on a constant rate replenishment strategy. In the early stage of fermentation, glucose was consumed at a rapid rate. The supplemented medium was added when the glucose concentration was below 2 g/L (12 h) and the growth was in an upward trend, but no significant increase was observed for the enzyme activity. It was thought that the supplemented glucose was mainly used for the growth of the strain. With the replenishment found that the overall rate of glucose consumption was on a downward trend, the growth of the strain also tended to level off, and the enzyme activity also decreased. The highest NADH pyrophosphatase activity reached 33 U/mL at 33 h, which was 3.5-fold higher than that of the flask level (after induction optimization) (Figure 9A). SDS-PAGE analysis indicated that the expression level of *EcNudc-M* was further enhanced (Figure 9B).

### 3.8 Biosynthesis of NMNH by mutant *EcNudc-M*

**FIGURE 10:**
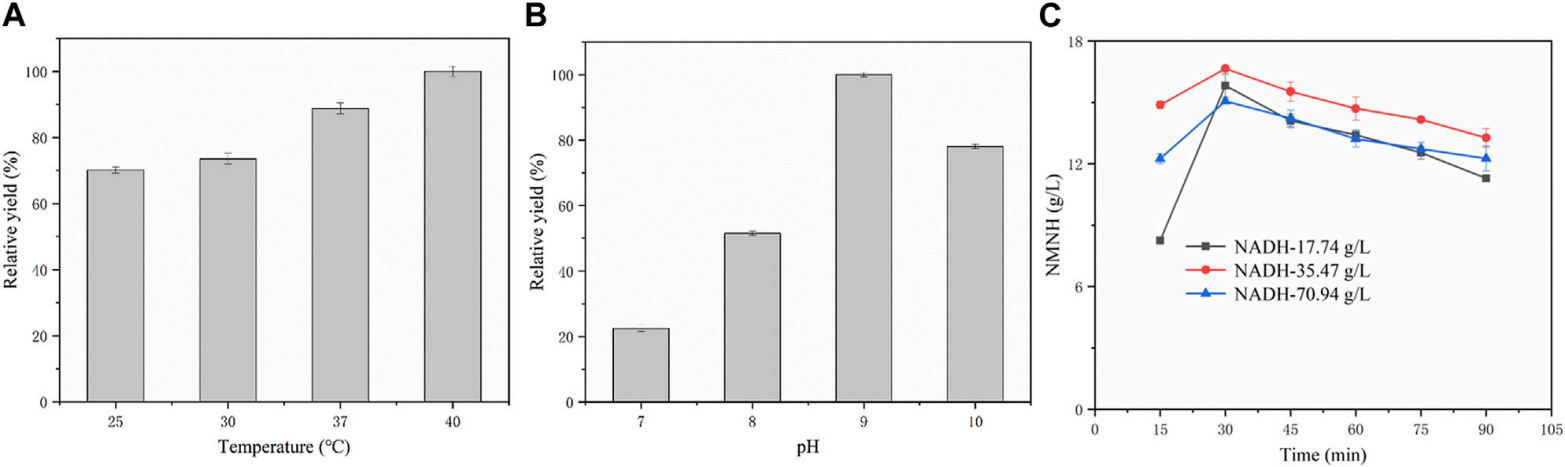
Exploration of catalytic process and biosynthesis of NMNH. **(A)** Relative yields of NMNH at different reaction temperatures. **(B)** Relative yields of NMNH at different reaction pH. **(C)** Biotransformation curve of NMNH at different substrate concentrations.

In order to evaluate the application of NADH pyrophosphatase for catalytic production of NMNH, the crude enzyme of *EcNudc-M* was used as a catalyst for biocatalytic reactions. However, in order to fully understand the catalytic properties of NADH pyrophosphatase recombinant bacteria on the substrate NADH, the optimal conditions for the catalytic reaction were first explored. The results are shown in Figure 10A, with the increase of reaction temperature, the NMNH yield also gradually increased, and the highest yield was reached at 40°C. pH is also an important factor affecting the catalytic reaction, as shown in Figure 10B, the conversion reaction had higher yield under alkaline conditions, in which, the highest yield was reached at pH = 9. Therefore, the best biotransformation experiment was performed at 40°C in pH = 9 buffer.

The biotransformation reactions were studied at 25 mM, 50 mM, and 100 mM substrate concentrations, respectively. The time courses of the bioconversion of NADH to NMNH were shown in Figure 10C. The highest yield of 16.65 g/L was achieved at 30 min at a substrate concentration of 50 mM and the conversion rate was 99.1%. However, after a second batch of substrate was put in at 30 min, the NMNH yield decreased, which is due to the instability of NMNH (Liu et al., 2021). When the substrate concentration was 25 mM, the highest yield reached 15.82 g/L and the conversion rate was 94.2% at 30 min (the second batch of substrate was put in at 15 min). However, the yield of NMNH also decreased after the third batch of substrate was put in at 30 min. When the substrate concentration was 100 mM, the yield reached a maximum of 15.06 g/L and the conversion rate was 44.8%. Therefore, the maximum yield of *EcNudc-M* in this study could be achieved at a substrate concentration of 50 mM, which is the highest level reported so far and further lays the foundation for industrial application.

## 4 Conclusion

NMNH has received substantial attention due to stimulating a rapid increase in NAD^+^ levels and its potential for applications such as the treatment of acute kidney injury. However, there are few studies on the enzymatic catalytic production of NMNH. Herein we successfully constructed an *E. coli* system for the efficient expression of NADH pyrophosphatase. The enzyme activity of NADH pyrophosphatase was significantly improved from 1.1 U/mL to 6.0 U/mL *via* expression regulation strategy and site-directed saturation mutagenesis. Furthermore, the thermal stability of *EcNudc-M* was also improved at 50°C by 3.6-folds. The molecular docking results showed that the weakened electrostatic interaction and the formation of hydrogen bonds were responsible for the enhancement of enzyme activity and thermal stability, respectively. The highest activity reached 33 U/mL by a continuous replenishment strategy in an expanded culture within a 7 L fermenter. Finally, the highest yield of NMNH was 16.65 g/L under the catalysis of *EcNudc-M*, which is the highest level reported so far. Overall, the mutant strain in this work improved the production efficiency of NMNH, which laid the foundation for its further industrial application.

## Data Availability

The datasets presented in this study can be found in online repositories. The names of the repository/repositories and accession number(s) can be found in the article/Supplementary Material.
