# Supplementary material for: Protein engineering of NADH pyrophosphatase for efficient biocatalytic production of reduced nicotinamide mononucleotide
Source: Front Bioeng Biotechnol. 2023 Apr 4;11:1159965. doi: 10.3389/fbioe.2023.1159965 (PMC10110983; doi:10.3389/fbioe.2023.1159965)
Supplement: Supplementary file 1 [file Table1.DOCX]

Supplementary Material

**Supplementary Table S1. The RBS primers used in this study.**

| **Primer name** | **Sequence (5'→3')** |
| --- | --- |
| RBS5000-F | TACTAACGTAAGGGGTCAGTTTAATGGGCAGCAGCCATCATCATC |
| RBS5000-R | TAAACTGACCCCTTACGTTAGTAATTATTTCTAGAGGGGAATTGTTAT |
| RBS7500-F | TATCCACCACCGCCATAAGGACTTCTAAAGTATGGGCAGCAGCCATCATCAT |
| RBS7500-R | ACTTTAGAAGTCCTTATGGCGGTGGTGGATAATTATTTCTAGAGGGGAATTG |
| RBS10000-F | GCATCAAAACCTACTCTATAAAGGGAATTATCATGGGCAGCAGCCATCATCATCA |
| RBS10000-R | GATAATTCCCTTTATAGAGTAGGTTTTGATGCATTATTTCTAGAGGGGAATTGTTA |
| RBS12500-F | GACACGACAGGGACATCAGAACTAAGGGTATTTATGGGCAGCAGCCATCATCATC |
| RBS12500-R | AAATACCCTTAGTTCTGATGTCCCTGTCGTGTCATTATTTCTAGAGGGGAATT |
| RBS15000-F | GAAGACACTCTACACTAACACATCGAGGCAAATAATGGGCAGCAGCCATCATCATCATC |
| RBS15000-R | TATTTGCCTCGATGTGTTAGTGTAGAGTGTCTTCATTATTTCTAGAGGGGAATTGTTAT |
| RBS17500-F | AGCGCAAGTTAATAAGTAGGTTCGCGACATGGGCAGCAGCCATCATCATC |
| RBS17500-R | GTCGCGAACCTACTTATTAACTTGCGCTATTATTTCTAGAGGGGAATTGT |
| RBS20000-F | TAAACAGTAACGATATTTAATAGGAAAATATGGGCAGCAGCCATCATCATCA |
| RBS20000-R | ATTTTCCTATTAAATATCGTTACTGTTTAATTATTTCTAGAGGGGAATTGTTATC |
| RBS22500-F | TTATCCGGCAAGAAAAGGCTTCGAAGCTTATGGGCAGCAGCCATCATCATC |
| RBS22500-R | AAGCTTCGAAGCCTTTTCTTGCCGGATAAATTATTTCTAGAGGGGAATTG |
| RBS25000-F | TCTCAATTTACAGACGGGGCCAATTTTTATGGGCAGCAGCCATCATCATC |
| RBS25000-R | AAAAATTGGCCCCGTCTGTAAATTGAGAATTATTTCTAGAGGGGAATTGTTAT |
| RBS30000-F | TTTCGACGAGGAAATAAAGGAGTTATAAGACAATGGGCAGCAGCCATCATCATCATC |
| RBS30000-R | TGTCTTATAACTCCTTTATTTCCTCGTCGAAAATTATTTCTAGAGGGGAATTGTTAT |
| RBS40000-F | TCCCGATCAGAACTTGGACCAGGTTTAAATGGGCAGCAGCCATCATCATC |
| RBS40000-R | TTAAACCTGGTCCAAGTTCTGATCGGGAATTATTTCTAGAGGGGAATTGT |
| RBS50000-F | ATACATCAAGTATAAAAGGGGTCTCATATGGGCAGCAGCCATCATCATCA |
| RBS50000-R | ATGAGACCCCTTTTATACTTGATGTATATTATTTCTAGAGGGGAATTGT |

Underline indicates the RBS sequence.


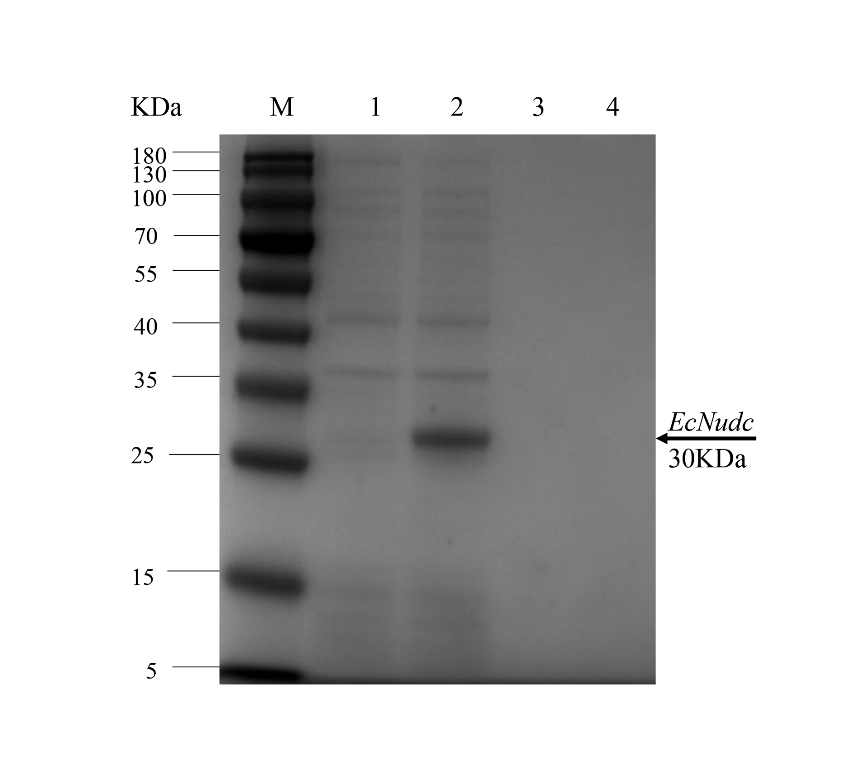


**Supplementary Figure S1**. SDS-PAGE analysis of recombinant *EcNudc* expression in host BL21(DE3). M: protein standard marker; Lane 1: the supernatant from *E. coli* BL21 (DE3) carrying pRSFDuet-1 plasmid; Lane 2: the supernatant of strain carrying plasmid pRSFDuet-EcNudc; Lane 3: the crush precipitation of strain carrying plasmid pRSFDuet-1; Lane 4: the crush precipitation of strain carrying plasmid pRSFDuet-EcNudc.


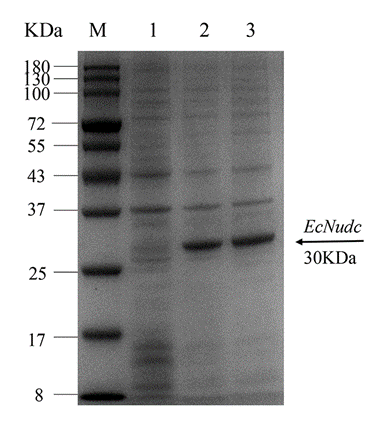


**Supplementary Figure S2**. SDS-PAGE analysis of recombinant *EcNudc* expression in host BL21(DE3). M: protein standard marker; Lane 1: the supernatant from *E. coli* BL21 (DE3) carrying pET-28 (+) plasmid; Lane 2: the supernatant of strain carrying plasmid pET-28a (+)-adhA+T7-RBS-EcNudc; Lane 3: the supernatant of strain carrying plasmid pET-28a (+)-adhA+T7-RBS-20000-EcNudc.


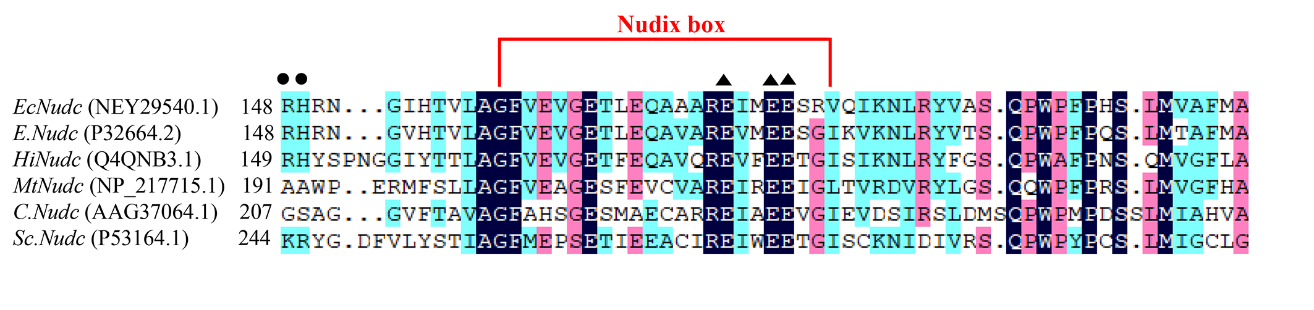


**Supplementary Figure S3**. Partial multiple sequence alignment of NADH pyrophosphatase. The sequence (NEY29540.1) was aligned with *Nudcs* from *Escherichia coli* K-12 (P32664.2), *Haemophilus influenza* (Q4QNB3.1), *Mycobacterium tuberculosis* (NP_217715.1), *Caenorhabditis elegans* (AAG37064.1), *Saccharomyces cerevisiae* (P53164.1). The conserved sequence GX_5_EX_7_REVXEEXGU was annotated. Catalytic residues annotated with positive triangles (▲). Mutant sites 148, 149 of *EcNudc* were marked with circles (●).


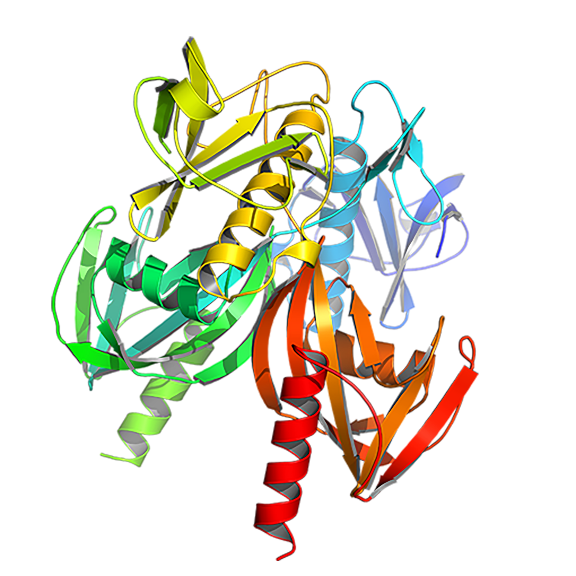


**Supplementary Figure S4**. Protein structure of *EcNudc*


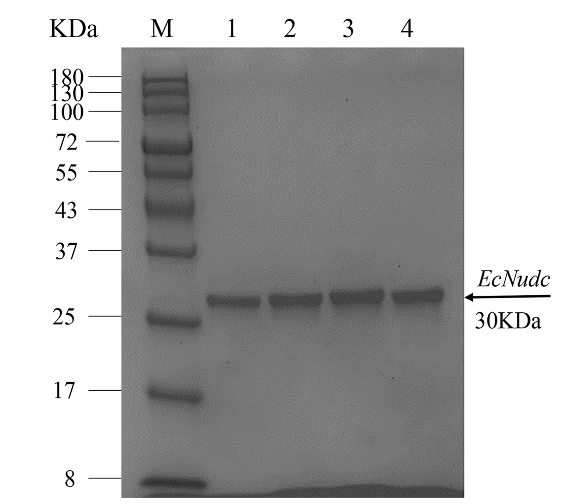


**Supplementary Figure S5**. SDS-PAGE analysis of *EcNudc* protein. M: protein standard marker; Lane 1: Purified wild type; Lane 2: Purified mutant R148A; Lane 3: Purified mutant H149E; Lane 3: Purified mutant *EcNudc-M.*
